# Numerical Simulation of Hybrid Electric–Structural Control for Microdroplet Formation in Ribbed T-Junction Microchannels

**DOI:** 10.3390/mi16070732

**Published:** 2025-06-22

**Authors:** Ruyi Fu

**Affiliations:** School of Science, Shanghai Institute of Technology, Shanghai 201418, China; fry_math@163.com

**Keywords:** microdroplet formation, ribbed T-junction, electric field, microdroplet size

## Abstract

Microdroplet formation in microfluidic systems plays a pivotal role in chemical engineering, biomedicine, and energy applications. Precise control over the droplet size and formation dynamics of microdroplets is essential for optimizing performance in these fields. This work explores a hybrid control strategy that combines an active electric field with passive rib structures to regulate the droplet formation in a ribbed T-junction microchannel under an electric field. Numerical simulations based on the phase-field method are employed to analyze the effects of the electric capillary number $Ca_{e}$ and rib height $a/w_{c}$ on the droplet formation mechanism. The results reveal that increasing $Ca_{e}$ induces three distinct flow regimes of the dispersed phase: unpinning, partially pinning, and fully pinning regimes. This transition from an unpinning to a pinning regime increases the contact area between the wall and dispersed phase, restricts the flow of the continuous phase, and induces the shear stress of the wall, leading to a reduction in droplet size with the enhanced $Ca_{e}$. Furthermore, an increase in rib height $a/w_{c}$ enhances the shear stress of the continuous phase above the rib, causing a progressive shift from a fully pinning to an unpinning regime, which results in a linear decrease in droplet size. A new empirical correlation is proposed to predict droplet size $S/w_{c}^{2}$ as a function of rib height $a/w_{c}$ and two-phase flow rate ratio $Q_{d}/Q_{c}$: $S/w_{c}^{2}=\left(-0.62-1.8Q_{d}/Q_{c}\right)\left(a/w\right)+(0.64+0.99Q_{d}/Q_{c})$.

## 1. Introduction

Microdroplets have garnered significant attention in chemical reactions [1,2,3], biological experiments [4,5,6], and materials science [7,8]. Their small size results in a larger surface-area-to-volume ratio, leading to faster reaction rates, making them indispensable tools in chemical processes. In biological applications, microdroplets serve as isolated containers for single cells or molecules, enabling efficient reactions within a confined space while preventing cross-contamination between reactions. Given the diverse droplet size requirements of microfluidic applications, the development of droplet generation technologies [9] and the precise control of microdroplet size have become critical areas of research. Microdroplet generation techniques are broadly categorized into passive [10] and active [11,12] control methods, primarily distinguished by the presence or absence of external energy input. Passive droplet generation techniques rely on channel geometry and hydrodynamic conditions to regulate droplet formation without the need for external energy input. These methods offer several advantages, including low cost, simple operation, and high throughput. Such characteristics make passive techniques ideal for applications such as microreactor construction, high-throughput analysis, and precision drug delivery. The common passive microdroplet generation methods include T-junction microchannels [13], flow focusing [14], and co-flow [15]. Among these, T-junction microchannels are widely utilized due to their simple structure, ease of fabrication, and high stability. Thorsen et al. [16] first investigated droplet formation in T-junction microchannels, demonstrating that droplet size is influenced by the microchannel geometry and internal pressure. Nisisako et al. [17] explored the effect of flow rate ratio on droplet generation in T-junction microchannels, observing that, as the two-phase flow rate ratio decreases, the droplet generation rate increases. Fu et al. [18] discovered that the droplet generation rate exhibited a nonlinear relationship with the two-phase flow rate ratio. Under fixed channel inlet width and flow rate ratio conditions, the droplet generation rate is linearly correlated with the dispersed-phase flow rate. Yao et al. [19] investigated the impact of different oil viscosities on water-in-oil emulsions, finding that the droplet size decreased with increasing oil viscosity across four different viscosity conditions, independent of flow pressure. Garstecki et al. [20] studied droplet and bubble formation in T-junction microchannels, concluding that, at low capillary numbers, droplet or bubble generation is controlled by the internal pressure drop rather than shear stress. Glawdel et al. [21] experimentally found that the droplet formation cycle in the dispersed phase includes an initial filling stage followed by a necking stage. Under the influence of interfacial tension, the dispersed phase retracts into the side channel during droplet formation. Xu et al. [22] examined the mechanism of droplet formation in T-junction microchannels, revealing that, as the capillary number of the continuous phase increases, droplets transition through three distinct flow regimes: squeezing, transient, and dripping. De Menech et al. [23] proposed that droplet formation in T-junction microchannels follows three different flow regimes: squeezing, dripping, and jetting. Among these, squeezing and dripping represent stable states, while jetting is predominantly unstable. Li et al. [24] conducted numerical simulations on multiphase flow in both conventional and modified T-junction microchannels. Their study found that the presence of ribs in T-junction microchannels can transition the jetting regime into a droplet regime while expanding the droplet regime region. Cui et al. [25] systematically investigated the dynamic interfacial behavior of surfactants in capillary-embedded stepped T-junction microchannels. Their research revealed that incorporating a stepped structure in the T-junction microchannel enhances interface instability and increases droplet generation frequency. Shen et al. [26] demonstrated that inserting a capillary tube at any desired location within a pre-fabricated microfluidic chip enables three-dimensional droplet generation. This innovative approach significantly mitigates the interference of channel walls on the droplet interface, effectively reducing the frequency of satellite droplet formation. Through combined experimental and numerical investigations, Lv et al. [27] demonstrated that the integration of a constraint structure downstream of the T-shaped microchannel effectively broadens the operational envelope for droplet formation control while significantly amplifying interfacial shear stress between the continuous ($\phi_{c}$) and dispersed ($\phi_{d}$) phases.

Active droplet generation technology achieves higher precision in microdroplet control by introducing external force fields to balance the various forces acting at the two-phase interface. The commonly applied external fields include magnetic fields [28,29,30], electric fields [31,32,33], temperature fields [34,35], and acoustic waves [36,37]. However, temperature and acoustic wave control often require long adjustment times, and magnetic fields can introduce fluid contaminate. As a result, active microdroplet control is typically achieved through the application of electric fields. Eribol and Uguz [38] applied an electric field in a rectangular microchannel to induce interfacial instability between two immiscible liquids. Their experimental results showed that increasing the viscosity ratio and flow rate ratio stabilizes the system. Kim et al. [39] utilized an electric field to control emulsion droplet formation in a flow-focusing microfluidic device, observing that droplet size decreased with increasing voltage. This method successfully reduced the droplet size to below 1 μm. Shojaeian and Hardt [40] experimentally investigated the effect of an electric field on the droplet size in a T-junction microchannel, demonstrating that an electric field significantly reduces the droplet size, suggesting its potential for the active control of individual droplet sizes, enabling the generation of sequential droplet flows. Altundemir et al. [41] experimentally applied a DC electric field perpendicular to the fluid flow and systematically studied the effects of thickness ratio, viscosity ratio, and total flow rate on droplet generation under different voltages. Singh et al. [42] conducted systematic experimental research on droplet formation under an electric field in a T-junction microchannel. Their study concluded that the presence of an electric field altered the droplet formation mechanism, causing the dispersed phase to emerge earlier in the main channel and fully block the entire channel. The application of an electric field resulted in reduced droplet size. Liu et al. [43] investigated the characteristics of droplet formation in a T-junction microchannel under an electric field, focusing on the effects of capillary number, flow rate ratio, and viscosity ratio on droplet size. Their findings revealed that, after applying an electric field, droplet flow appeared in the flow field at lower capillary numbers. The droplet size decreased as the continuous phase velocity and viscosity ratio increased. Li et al. [44] conducted numerical studies to summarize the behavior of dispersed-phase fluids with different viscosities, concluding that the presence of an electric field shifted the microdroplet generation mechanism from shear-dominated to squeezing-dominated, thereby increasing the droplet generation rate. At high viscosity ratios, increasing the capillary number promotes droplet coalescence. Wang et al. [45] designed a coaxially aligned capillary microchannel to study the effect of the electric field on droplet formation and found that droplet size decreased with increasing continuous phase flow rate and voltage, while the formation time of droplets decreased with increasing dispersed-phase flow rate and voltage. In passive-controlled microdroplet generation, incorporating active control enables precise regulation of droplet size without altering the intrinsic microchannel structure. Conversely, introducing passive control into active-controlled microdroplet generation can surpass the limitations of purely active manipulation on droplet formation, thereby achieving further reduction in droplet dimensions. This synergistic integration of dual control mechanisms demonstrates complementary advantages for optimizing microfluidic droplet production.

In summary, the body of research on the formation mechanism of microdroplets in a T-junction microchannel, adopting a hybrid passive and active control approach, remains scarce. Compared to a single control method, the combination of active and passive approaches allows for better regulation of droplet formation in microchannels. In this paper, a hybrid approach that integrates electric field control with a passive rib structure is used to investigate the influence of electric capillary number and rib height on the droplet formation dynamics in a T-junction microchannel. The phase-field method is used to simulate the two-phase behavior, analyzing the droplet formation mechanism under the influence of an electric field in a modified ribbed T-junction microchannel. The effects of different electric capillary numbers and rib heights on the dispersed-phase flow patterns are also examined. Finally, the impact of parameters such as flow rate ratio, viscosity ratio, rib height, and electric capillary number on droplet size is analyzed. This study provides a theoretical basis for droplet formation in ribbed T-junction microchannels under electric fields, offering insights into methods for producing small-scale droplets and achieving precise droplet size control in microfluidic mechanics.

## 2. Numerical Methods

### 2.1. Governing Equations

In the T-junction microchannel, the channel dimensions are on the order of micrometers, and the flow within the device is considered laminar. The two fluid phases are immiscible and incompressible Newtonian fluids. The continuity equation and momentum equation can be expressed as

(1) ∇·u=0

(2) $$\rho \frac{\partial u}{\partial t}+\rho \left(u\;\cdot \;\nabla \right)u=-\nabla p+\nabla \left[\mu \left(\nabla u+{\left(\nabla u\right)}^{T}\right)\right]+F_{\gamma }+F_{e}$$

where ρ (kg/m^3^) is the density, u (m/s) is the velocity, *t* (s) is the time, *p* (Pa) is the pressure, μ (Pa·s) is the viscosity, $F_{\gamma }$ (N/m^3^) is the momentum source term due to interfacial tension, and $F_{e}$ (N/m^3^) is the momentum source term due to the electric field force. The interfacial tension between the two phases is calculated as

(3) $$F_{\gamma }=G\nabla \phi$$

where *G* (J/m^3^) is the chemical potential, expressed as

(4) $$G=\lambda \left[-\nabla^{2}\phi +\frac{\phi (\phi^{2}-1)}{\epsilon^{2}}\right]$$

In the droplet generation process in a ribbed T-junction microchannel under the electric field, accurately tracking the dynamic behavior of the deformation and rupture of the two-phase interface is a key focus of this study. The phase field method, based on the Cahn–Hilliard equation, is used to describe the evolution of the liquid–liquid interface. This method introduces the phase field function ϕ to track the liquid–liquid interface, and ϕ is solved from the Cahn–Hilliard free energy equation:

(5) $$\frac{\partial \phi }{\partial t}+u\;\cdot \;\nabla \phi =\nabla \;\cdot \;\gamma \nabla G$$

The Cahn–Hilliard Equation (5) is typically decomposed into Equations (6) and (7), which are used to solve the phase field variable and the mixed energy density, respectively, to determine the position of the liquid–liquid interface.

(6) $$\frac{\partial \phi }{\partial t}+u\;\cdot \;\nabla \phi =\nabla \;\cdot \;\frac{\gamma \lambda }{\epsilon^{2}}\nabla \psi$$

(7) $$\psi =-\nabla \;\cdot \;\left(\epsilon^{2}\nabla \phi \right)+\left(\phi^{2}-1\right)\phi$$

where γ (m^3^s/kg) is the mobility coefficient, λ (N) is the mixing energy density, and ϵ (m) is a numerical parameter that controls the characteristic length-scale of the thin transition layer. The continuous phase is defined as ϕ=−1, the dispersed-phase value as ϕ=1, and the interface value as −1<ϕ<1. Therefore, the volume fraction of the continuous phase and the dispersed phase are defined as

(8) $$V_{f1}=\frac{1-\phi }{2}$$

(9) $$V_{f2}=\frac{1+\phi }{2}$$

Thus, the density, viscosity, dielectric constant, and conductivity at the interface can be expressed as

(10) $$\rho_{i}=\rho_{c}V_{f1}+\rho_{d}V_{f2}$$

(11) $$\mu_{i}=\mu_{c}V_{f1}+\mu_{d}V_{f2}$$

(12) $$\epsilon_{i}=\epsilon_{c}V_{f1}+\epsilon_{d}V_{f2}$$

(13) $$\sigma_{i}=\sigma_{c}V_{f1}+\sigma_{d}V_{f2}$$

The electric field force is calculated by solving the electric field distribution based on the droplet’s position and shape. The electric field force is then introduced as a volumetric force in Equation (2). It is assumed that free charges and magnetic effects are neglected, and the fluid is treated as a perfect dielectric material. The Maxwell equations govern the distribution of the electric field:

(14) $$\nabla \left(\epsilon E\right)=0,\;\epsilon =\epsilon_{0}\epsilon_{i}$$

where $\epsilon_{0}$ (F/m) is the vacuum permittivity, $\epsilon_{i}$ is the relative dielectric constant at the two-phase interface calculated, and *E* is the electric field intensity. The electric field intensity is calculated using the equation E=−∇U, where *U* (V) is the electric potential. Maxwell’s Equation (14) can be transformed into

(15) −∇·(ϵ∇U)=0

After solving for the electric field intensity, the electric displacement $D=\epsilon_{0}\epsilon_{i}E$. The divergence of the Maxwell stress tensor determines the electric field force:

(16) $$M=ED-\frac{1}{2}\left(E\;\cdot \;D\right)I$$

(17) $$F_{e}=\nabla \;\cdot \;M=qE-\frac{1}{2}E^{2}\nabla \left(\epsilon_{0}\epsilon_{i}\right)$$

Under the condition of no free charge (q=0) and incompressible fluid, Equation (17) can be rewritten as

(18) $$F_{e}=-\frac{1}{2}E^{2}\nabla \left(\epsilon_{0}\epsilon_{i}\right)$$

Recent advances in multiphysics modeling have demonstrated the effectiveness of COMSOL Multiphysics 6.2 in simulating coupled physical phenomena. In this study, this computational platform is employed to investigate electric field-mediated droplet generation within a ribbed T-junction microchannel. The numerical framework integrates laminar flow dynamics with a phase-field approach from the software’s CFD module to capture two-phase interfacial phenomena, while the electrostatic distribution is resolved through the solution of the Poisson equation using the AC/DC module’s electrostatics interface.

### 2.2. T-Junction Microchannel Geometry

**Figure 1:**
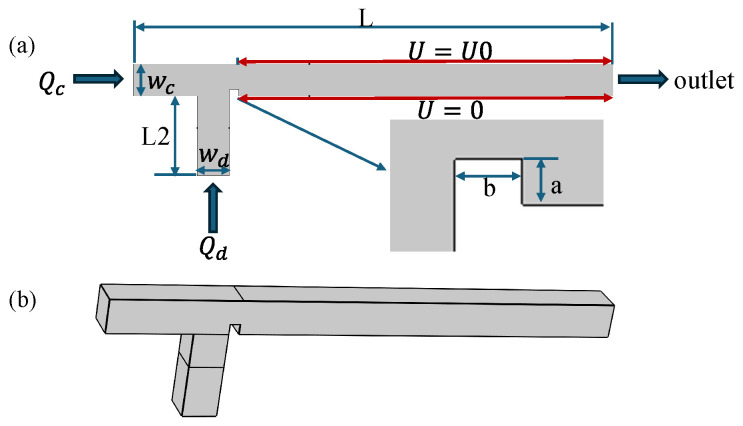
(**a**) Schematic of a ribbed T-junction microchannel under an electric field. Inlet figure: a schematic diagram of a rib with the width of *b* and height of *a*. (**b**) 3D schematic diagram.

The geometric configuration of the ribbed T-junction microchannel is illustrated in Figure 1. As shown in Figure 1, ribs have been added downstream of the T-junction microchannel. Compared to traditional T-junction microchannel, the rib structure can enhance the shear effect on dispersed-phase fluids, thereby expanding the range of droplet formation and increasing the frequency of droplet formation. Electrodes are positioned on the upper wall of the main channel and connected to a high-voltage power supply, while corresponding electrodes on the lower wall are grounded. A potential difference U(V) is generated between these electrodes, enabling active control of microdroplet formation. A rectangular rib with the width of *b* and height of *a* is placed at the junction of the two channels to achieve passive control of microdroplet formation. The wall boundary condition is set as a non-slip wetted wall. The channel thickness is set to 100 μm. The continuous phase (CP) enters from the main channel inlet, while the dispersed phase (DP) enters from the side channel inlet. The outlet pressure is set to 0 Pa. The fluid parameters chosen for the simulation are referenced from the study conducted by Liu et al. [43]. Detailed channel parameters and physical properties of the two phases are listed in Table 1. Generally, the microchannel droplet formation process is influenced by parameters such as the surface tension coefficient γ, dynamic viscosities of the dispersed and continuous phases $\mu_{d}$ and $\mu_{c}$, flow rates of two phases $Q_{d}$ and $Q_{c}$, as well as the effects of electric fields when applied. The dimensionless forms of these parameters are as follows: $Q=\frac{Q_{c}}{Q_{d}}$, $\lambda =\frac{\mu_{d}}{\mu_{c}}$, $Ca=\frac{Q_{c}\mu_{c}}{\gamma }$, $Ca_{e}=\frac{\varepsilon_{0}\varepsilon_{c}w_{c}E^{2}}{2\gamma }$.

## 3. Model Validation

### 3.1. Grid Independence Verification

**Figure 2:**
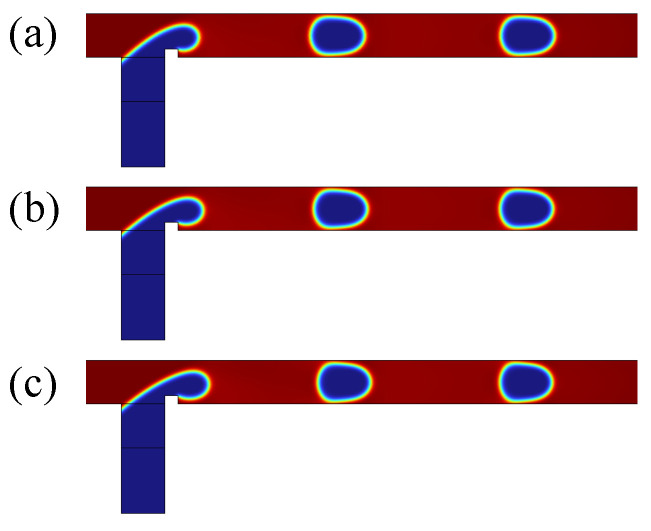
Effect of grid size on the volume fraction of the dispersed phase: (**a**) grid size 9843; (**b**) grid size 13,105; (**c**) grid size 18,468.

A grid independence study was conducted to ensure the accuracy and reliability of the numerical simulations. Three different grid sizes were used, the number of grid elements being 9843, 13,105, and 18,468. Figure 2 illustrates the distribution of the volume fraction of the dispersed phase at the same time. The calculated areas of the dispersed-phase droplets for these three grids are 8662 μm^2^, 8484 μm^2^, and 8427 μm^2^, respectively. It is evident that the droplet sizes obtained with the latter two grid sizes are nearly identical, indicating that the results are grid-independent beyond 13,105 elements.

**Figure 3:**
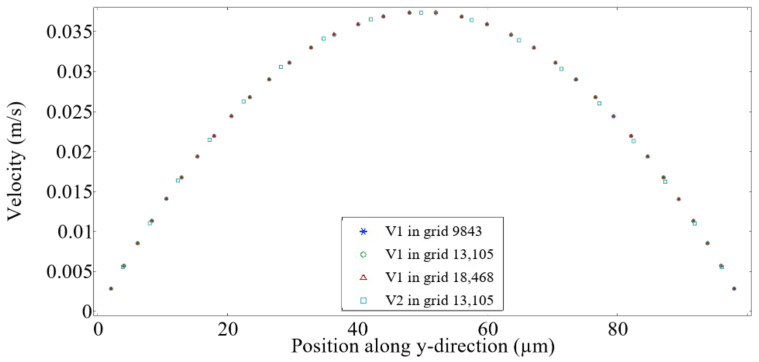
Grid independence study with $Q_{d}=0.3$ mL/h and $Q_{c}=0.9$ mL/h. $V_{1}$ and $V_{2}$ represent the velocity along the y-direction at x=100 μm and x=150 μm, respectively.

As shown in Figure 3, the velocity distributions along the y direction are presented for three grid sizes at two different locations, x=100 μm ($V_{1}$) and x=150 μm ($V_{2}$). The velocity profiles for $V_{1}$ show negligible differences between the three grid sizes, confirming that the flow field is adequately resolved with 13,105 elements. Furthermore, the velocity $V_{1}$ at x=100 μm closely matches the velocity $V_{2}$ at x=150 μm, indicating that the flow field is fully developed 100 μm downstream of the inlet. Therefore, a triangular grid with 13,105 elements was selected to optimize computational efficiency for all subsequent simulations.

### 3.2. Data Validation

**Figure 4:**
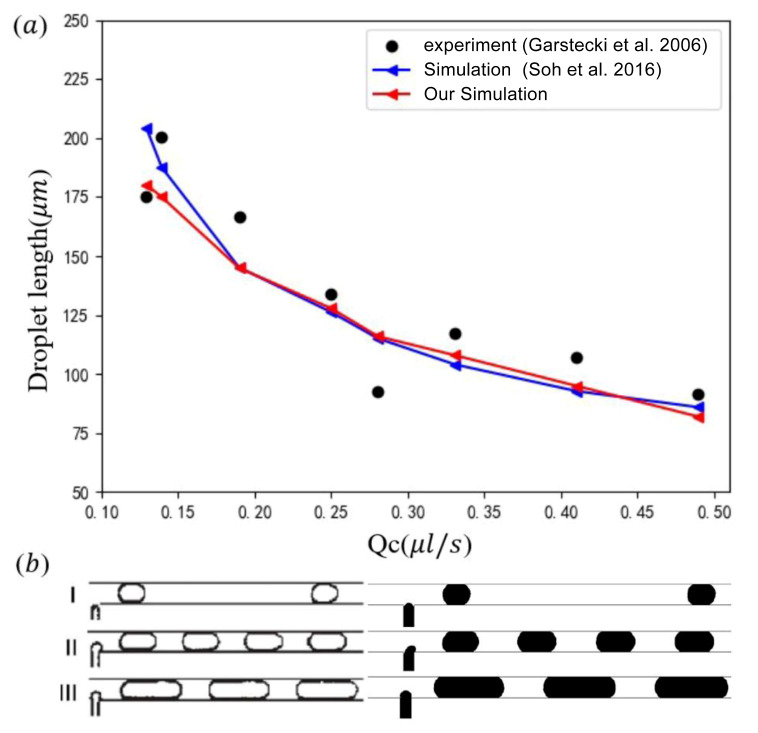
(**a**) Comparison of the droplet length from the experiment [20] and simulation [46] at $Q_{d}=0.14\;\mu L\;s^{-1}$ under different $Q_{c}$; (**b**) visualization of droplet formation from experiments [20] (left) and our simulations (right) under (I) $Q_{d}=0.004\;\mu L\;s^{-1}$, $Q_{c}=0.028\;\mu L\;s^{-1}$; (II) $Q_{d}=0.14\;\mu L\;s^{-1}$, $Q_{c}=0.139\;\mu L\;s^{-1}$; (III) $Q_{d}=0.111\;\mu L\;s^{-1}$, $Q_{c}=0.028\;\mu L\;s^{-1}$.

To validate the reliability of the current model, we considered two cases: the droplet motion in a T-junction microchannel and the deformation of a static droplet under an electric field. The experimental data from Garstecki et al. [20] were used to validate the dynamic behaviors of microdroplets. The geometric and physical parameters of the T-junction microchannel used for validation are summarized in Table 2. The widths of the main channel and side channels are 100 μm and 50 μm, respectively, with a uniform depth of 33 μm. Figure 4 provides a quantitative comparison between our numerical results, the experimental results [20], and the simulation results from Soh et al. [46]. As the flow rate of the continuous phase increases, the droplet size gradually decreases. While experimental results are susceptible to external disturbances, leading to fluctuations, the numerical simulations eliminate such noise, resulting in smoother trends. Minor discrepancies between our simulations and those of Soh et al. [46] are attributed to differences in numerical methods. However, the overall agreement is excellent, and the droplet morphology and size calculated under three different operating conditions align well with the experimental observations in Figure 4. Obviously, this confirms the reliability of the flow field simulations in this study.

**Figure 5:**
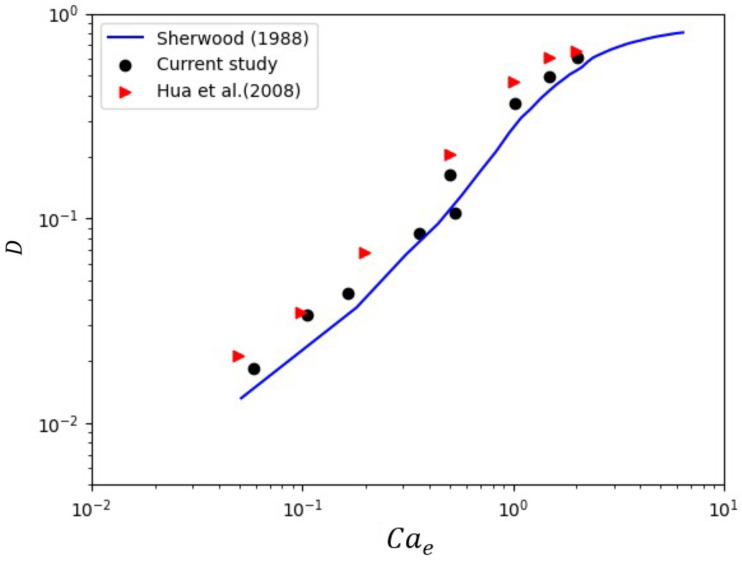
Comparison of the relationship of the deformation parameter and electrocapillary number between the current method and those of Sherwood [47] and Hua et al. [48].

The deformation of a stationary droplet under an electric field was simulated to validate the numerical accuracy of the coupled electric field and two-phase flow model. A circular droplet with a radius *R* was placed in a computational domain of 6R×6R, with the top boundary maintained at a high electric potential and the bottom boundary grounded. Figure 5 shows the relationship between the deformation parameter *D* and the electrocapillary number $Ca_{e}$. *D* is defined as (A−B)/(A+B), where *A* and *B* represent the major and minor axes of the deformed droplet, respectively. The simulation results were compared with theoretical predictions from Sherwood [47] and numerical results from Hua et al. [48]. As shown in Figure 5, the current results exhibit excellent agreement with both the analytical and numerical predictions, confirming the accuracy of the coupled model. These validation steps demonstrate the robustness and reliability of the numerical framework employed in this study, providing a solid foundation for the subsequent analysis of droplet generation mechanisms in ribbed T-junction microchannels under electric fields.

## 4. Results and Discussion

### 4.1. The Droplet Formation Mechanism

**Figure 6:**
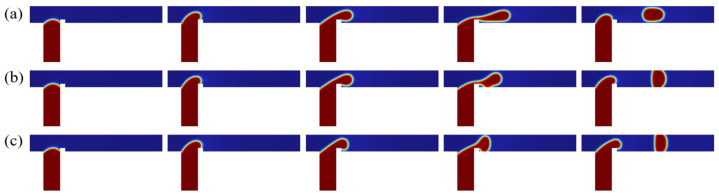
Time evolution of the dispersed-phase volume fraction distribution at three electric capillary numbers (**a**) ${\mathrm{Ca}}_{e}=0$, (**b**) ${\mathrm{Ca}}_{e}=0.38$, and (**c**) ${\mathrm{Ca}}_{e}=1.14$, where Ca=0.036, Q=3, $a/w_{c}=0.2$, and $b/w_{c}=0.3$.

**Figure 7:**
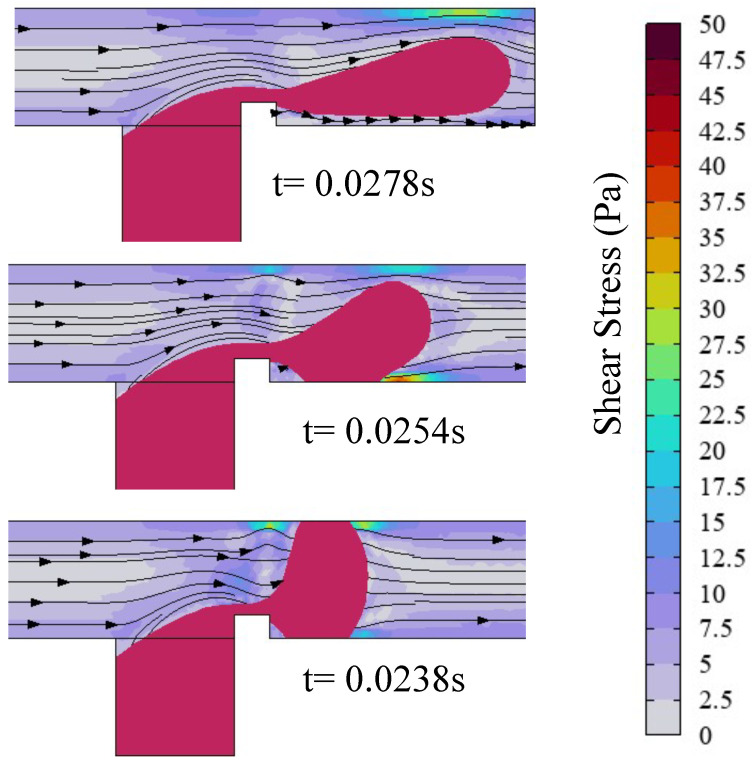
Shear stress and streamline distribution in the microchannel, with the red line representing the isosurface of the dispersed-phase volume fraction (ϕ=0.5), where Ca=0.036, Q=3, $a/w_{c}=0.2$, and $b/w_{c}=0.3$. ${\mathrm{Ca}}_{e}=0$ (up), ${\mathrm{Ca}}_{e}=0.38$ (middle), and ${\mathrm{Ca}}_{e}=1.14$ (down).

Understanding microdroplet morphology is of great significance for elucidating its formation mechanism. Firstly, the flow regimes of a discrete phase in a ribbed T-junction microchannel under an electric field are investigated. Figure 6 illustrates the effect of different electric fields on the dispersed-phase flow pattern in a ribbed T-junction microchannel under capillary number Ca=0.036 and flow ratio Q=3. By systematically increasing the electric capillary number $Ca_{e}$, we examined the dispersed-phase behavior while maintaining a constant rib height. Since the electric field is applied downstream of the ribs, the initial motion of the dispersed phase entering the main channel remains unaffected by the electric field, as demonstrated by comparing three different cases (${\mathrm{Ca}}_{e}=0$, 0.38, and 1.14). Upon entering the main channel from the side channel, the dispersed phase accumulates upstream of the rib due to flow obstruction. This accumulation process causes the dispersed phase to detach from the wall after passing over the rib and ceases to adhere to the channel wall. As shown in Figure 6a, for the ${\mathrm{Ca}}_{e}=0$ case, the dispersed phase detaches from the wall after crossing the rib, forming an unpinning flow pattern. As it moves downstream, necking and rupture occur, resulting in droplet formation. In Figure 6b,c, after the electric field is applied, the dispersed phase is stretched in the y-direction. As shown in Figure 6b, for the ${\mathrm{Ca}}_{e}=0.38$ case, after crossing the rib, the dispersed phase gradually deforms under the electric field and moves downstream while remaining attached to the bottom wall of the channel, forming a partially pinning flow. The resistance of the wall slows the downstream motion of the dispersed phase. As shown in Figure 6c, for ${\mathrm{Ca}}_{e}=1.14$, the dispersed phase experiences a stronger electric field force. After passing over the rib, the dispersed phase completely blocks the entire channel under the influence of the electric field and attaches to both the upper and lower walls of the main channel, forming a fully pinning flow pattern. As can be seen from Figure 7, the isosurface of the discrete phase volume fraction under three different $Ca_{e}$ clearly reveals three distinct flow patterns: unpinning, partially pinning, and fully pinning.

A substantial body of research has demonstrated that, in T-junction microchannels, the formation of dispersed-phase droplets is governed by the shear force of the continuous phase and the pressure drop inside the channel. Liu’s study [43] revealed that applying an electric field induces the deformation of the dispersed phase, thereby modulating both the continuous-phase shear forces and the pressure dynamics of the channel. The microdroplet formation mechanism is investigated through analysis of the effect of the electric capillary number ${\mathrm{Ca}}_{e}$ on the shear force of the continuous phase and the channel pressure drop in a ribbed T-junction microchannel. As shown in Figure 7, for the case of ${\mathrm{Ca}}_{e}=0$, the ribs lift the dispersed phase away from the main channel walls, preventing direct contact. Consequently, the dispersed phase experiences negligible wall shear force, leading to prolonged downstream stretching and an extended necking/breakup duration (t=0.0275 s). For the case of ${\mathrm{Ca}}_{e}=0.38$, upon passing the ribs, the dispersed phase undergoes y-axis stretching under the electric field, causing adhesion to the bottom wall of the main channel. This contact introduces shear stress from the wall, thereby reducing the downstream stretching length. Moreover, electric field-driven upward stretching of the dispersed phase narrows the cross-sectional area near the upper wall, accelerating the continuous phase flow. This constriction amplifies the shear stress exerted by the continuous phase on the dispersed-phase interface. These combined effects enhance droplet formation frequency while reducing droplet size. At ${\mathrm{Ca}}_{e}=1.14$ in Figure 7, significantly enhanced electric field forces rapidly stretch the dispersed phase to fully block the channel after rib passage. The continuous phase, obstructed by the dispersed phase, redirects its flow and generates a vortex at the upper-left interface of two-phase flow. This flow redirection intensifies shear stress on the dispersed phase. Furthermore, simultaneous contact with both the upper and lower channel walls strengthens the shear resistance from the walls, further weakening the downstream stretching. Under these conditions, droplet formation occurs at the higher frequency, producing the smaller droplet size. Obviously, as the electric capillary number increases, the degree of deformation of the dispersed phase becomes greater, increasing the shear stress of the continuous phase and accelerating the droplet formation frequency. The increased shear stress from the walls shortens the length of downstream stretching, reducing the droplet size.

**Figure 8:**
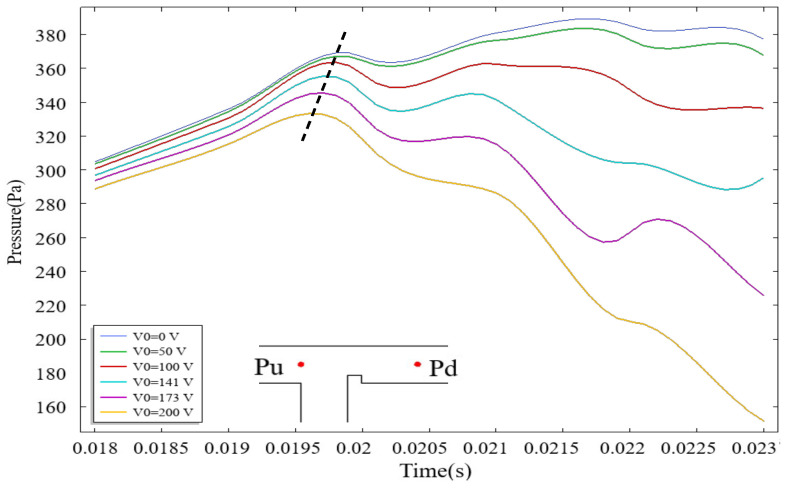
The variation in upstream pressure over time in the microchannel under different voltage conditions, where the dashed line represents the connection of the extreme value points, and the red dots indicate the locations of the upstream pressure points. Red markers denote measurement locations of $P_{u}$ and $P_{d}$.

Under the influence of an electric field, the pressure drop within the microchannel constitutes a critical additional factor governing droplet formation. To quantify the pressure drop in the channel, upstream point $P_{u}$ and downstream points $P_{d}$ were designated for pressure data acquisition, with the pressure drop $P_{u}-P_{d}$ calculated accordingly. Figure 8 illustrates the temporal evolution of upstream pressure $P_{u}$ in the microchannel under different ${\mathrm{Ca}}_{e}$, where red markers denote measurement locations of $P_{u}$ and $P_{d}$ within the T-junction geometry. Upon entering the main channel, the dispersed phase encounters rib-induced flow obstruction, leading to upstream accumulation and a progressive rise in $P_{u}$. Higher applied voltages intensify the interaction of the electric field with the dispersed phase, thereby reducing the time required for the dispersed phase to traverse the rib region. As depicted in Figure 8, increasing voltage correlates with two distinct trends: a reduction in peak $P_{u}$ magnitude and a shortened duration to attain peak pressure. These observations align with the pressure-driven dynamics reported by Singh et al. [42], suggesting a transition in the driving mechanism for dispersed-phase transport from upstream pressure dominance to electric field force dominance.

**Figure 9:**
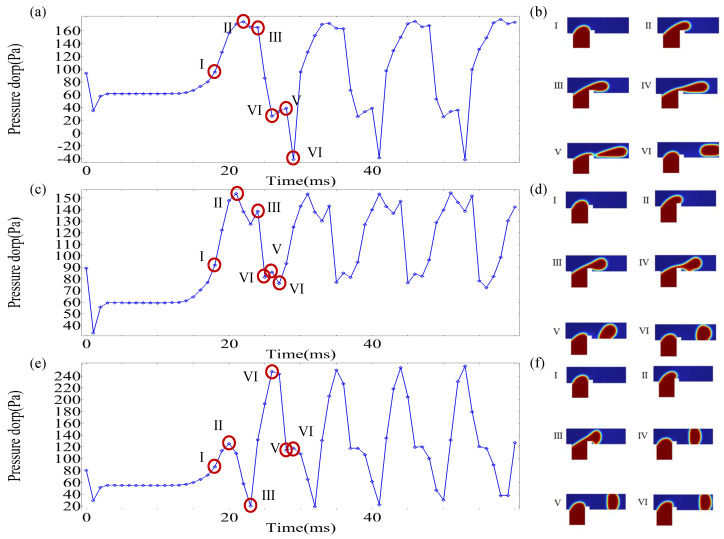
The evolution of the channel pressure drop over time under different electric capillary numbers ($Ca_{e}$) and the corresponding dispersed-phase morphology. (**a**,**b**) ${\mathrm{Ca}}_{e}=0$; (**c**,**d**) ${\mathrm{Ca}}_{e}=0.38$; (**e**,**f**) ${\mathrm{Ca}}_{e}=1.14$.

Figure 9 demonstrates the temporal evolution of channel pressure drop $P_{u}-P_{d}$ under varying ${\mathrm{Ca}}_{e}$ alongside corresponding flow-state snapshots at critical stages. The periodic generation of droplets induces cyclical pressure drop accumulation and release within the microchannel, reflecting two distinct stages of droplet formation. The rise in pressure drop indicates the filling stage, where the dispersed phase enters the main channel from the side channel, obstructing continuous phase flow. This obstruction enhances the driving force, ultimately leading to droplet formation via neck breakup of the dispersed phase. The pressure drop $P_{u}-P_{d}$ describes the squeezing stage, where the continuous phase displaces the dispersed phase downstream, promoting droplet pinch-off at the neck region. Concurrently, the pressure drop gradually diminishes as the dispersed phase advances. Stages I–II in Figure 9b,d,f correspond to the filling stage of the dispersed phase. With increasing ${Ca}_{e}$, the duration of this stage decreases from 4 ms to 3 ms, and further to 2 ms, indicating accelerated electric field-driven dispersion of the phase into the main channel. Consequently, higher voltages shorten filling-stage times. The pressure drop cycle, defined as the time interval between consecutive peaks in Figure 9a,c,e, provides a proxy for droplet production frequency. The calculated cycles measure 12 ms, 10 ms, and 9 ms for low, moderate, and high ${\mathrm{Ca}}_{e}$, respectively. It can be seen that, in T-junction microchannels without an electric field, the time required for droplet formation is the longest. After increasing the electric field, the droplet formation time is shortened, and, as the potential increases, the droplet formation time is shortened. After adding an electric field, the model is compared with passive T-junction mode, which can effectively shorten the droplet formation time and accelerate the droplet generation frequency.

**Figure 10:**
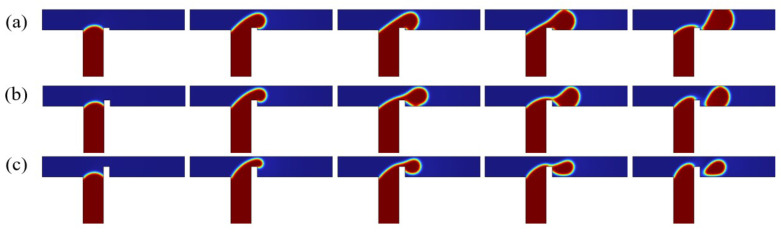
Evolution of dispersed-phase volume fraction over time for different rib heights, where Ca=0.02, Q=1.67, $Ca_{e}=0.38$, and $b/w_{c}=0.3$. $a/w_{c}=0.1$ (**a**); $a/w_{c}=0.3$ (**b**); $a/w_{c}=0.5$ (**c**).

In T-junction microchannels, channel geometry critically influences droplet formation dynamics. As illustrated in Figure 10, systematic variations in rib height and the constant electric field strength $Ca_{e}=0.38$ were employed to isolate the role of the rib in droplet generation.

As shown in Figure 10a, for the case of rib height $a/w_{c}=0.1$, the rib induces minor elevation of the dispersed phase, partially obstructing the continuous-phase flow. Downstream transport of the dispersed phase is accompanied by electric field-driven vertical (y-axis) stretching. Proximity to the lower wall initiates dispersed-phase adhesion, followed by upper wall attachment to form a fully pinning flow regime, culminating in complete channel occlusion. When the rib height $a/w_{c}=0.3$, as shown in Figure 10b, increased rib height elevates the dispersed phase, narrowing the upper channel cross-section and significantly impeding continuous-phase flow. Electric field forces drive dispersed-phase adhesion to the lower wall exclusively, establishing a partially pinning flow regime. For the rib height $a/w_{c}=0.5$ in Figure 10c, the rib elevation positions the dispersed phase near the upper wall without adhesion, maintaining an unobstructed flow path. Throughout the pre-pinch-off phase, the dispersed phase remains non-adherent to channel walls, sustaining an unpinning flow regime. Moreover, it can be obtained that, the higher the rib, the smaller the droplet size formed.

**Figure 11:**
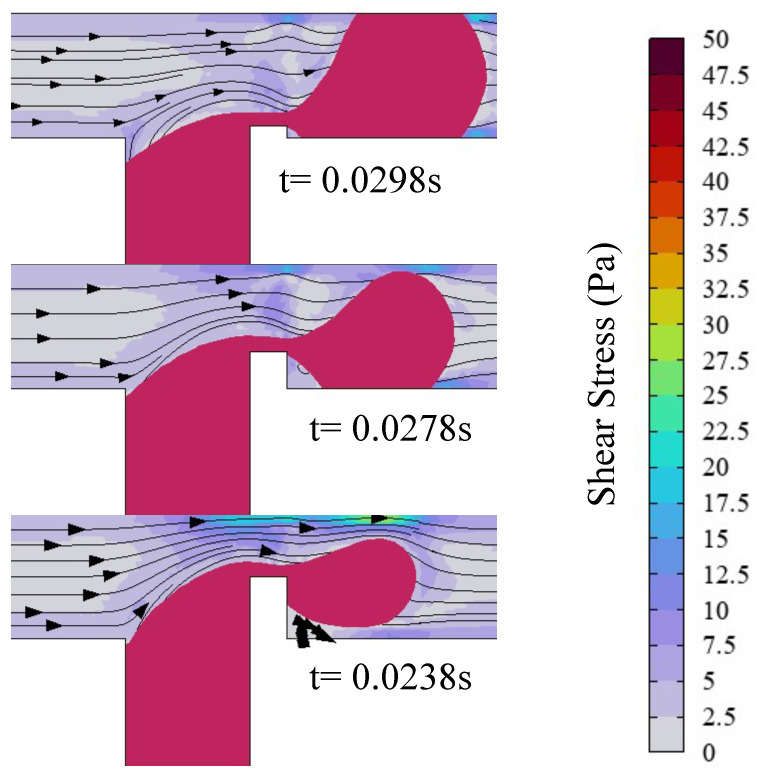
Shear stress and streamline distribution in the microchannel, with the red line representing the iso-contour of dispersed-phase volume fraction (ϕ=0.5), where Ca=0.02, Q=1.67, $Ca_{e}=0.38$, and $b/w_{c}=0.3$. $a/w_{c}=0.1\;$(up); $a/w_{c}=0.3$ (middle); $a/w_{c}=0.5$ (down).

Figure 11 depicts the shear stress distribution and streamline profiles within the microchannel immediately preceding droplet formation for three rib height ratios $a/w_{c}=0.1,0.3,0.5$. For the case of $a/w_{c}=0.1$, the dispersed phase resides in a fully pinning flow regime prior to droplet pinch-off, experiencing pronounced shear resistance from both the upper and lower channel walls. The low rib height minimally obstructs continuous-phase flow, yielding attenuated shear stress at the dispersed-phase neck. At $a/w_{c}=0.3$, a partially pinning flow regime prevails before droplet formation, with shear resistance localized to the lower wall. Rib-induced flow obstruction accelerates the continuous-phase velocity above the rib, thereby amplifying shear stress at the neck region. This constriction accelerates the continuous phase near the droplet’s upper interface, intensifying interfacial shear stress and expediting necking dynamics. For the $a/w_{c}=0.5$ case, the dispersed phase operates in the unpinned flow regime, free from wall-induced shear resistance. The elevated rib height positions the dispersed phase closer to the upper channel region, amplifying the continuous-phase velocity above the rib and generating elevated shear stress at the neck. Simultaneously, the electric field-driven upward migration of the dispersed phase toward the upper wall further accelerates the continuous phase, intensifying shear stress at the droplet’s upper interface. This dynamic suppresses sustained adhesion of the dispersed phase to the upper wall. As the rib height ratio increases, the shear stress of the continuous phase above the rib becomes larger, causing the flow regime of the dispersed phase to transition from a fully pinning flow state to a partially pinning flow state and eventually to an unpinning flow state. Concurrently, heightened shear stress accelerates the droplet formation kinetics and reduces droplet size.

**Figure 12:**
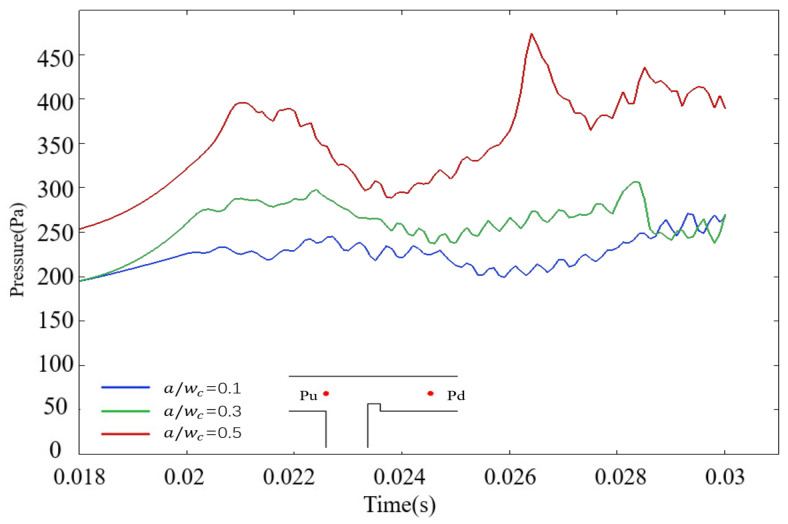
The graph of upstream pressure variation over time under different rib height ratios in the microchannel. The blue line represents the case of $a/w_{c}=0.1$; the green line represents the case of $a/w_{c}=0.3$; the red line represents the case of $a/w_{c}=0.5$.

Subsequently, rib-induced modifications to droplet dynamics are analyzed via pressure field measurements in a ribbed T-junction microchannel under an electric field. During the filling stage, the dispersed phase enters the main channel and accumulates at the rib’s top end, leading to a progressive rise in upstream pressure. As the rib height ratio $a/w_{c}$ increases, the maximum upstream pressure escalates correspondingly. As shown in Figure 12, during the filling stage, when the rib height ratio $a/w_{c}=0.1$, the maximum pressure within the T-junction microchannel during the filling stage measures 226 Pa. When the rib height ratio increases to $a/w_{c}=0.3$, the peak pressure rises to 288 Pa, reflecting a 27.4% increase compared to that of $a/w_{c}=0.1$. For $a/w_{c}=0.5$, the maximum pressure surges to 396 Pa, marking a 75.2% increase relative to the baseline case ($a/w_{c}=0.1$). These results underscore a strong positive correlation between rib height ratio and upstream pressure magnitude during the filling stage.

**Figure 13:**
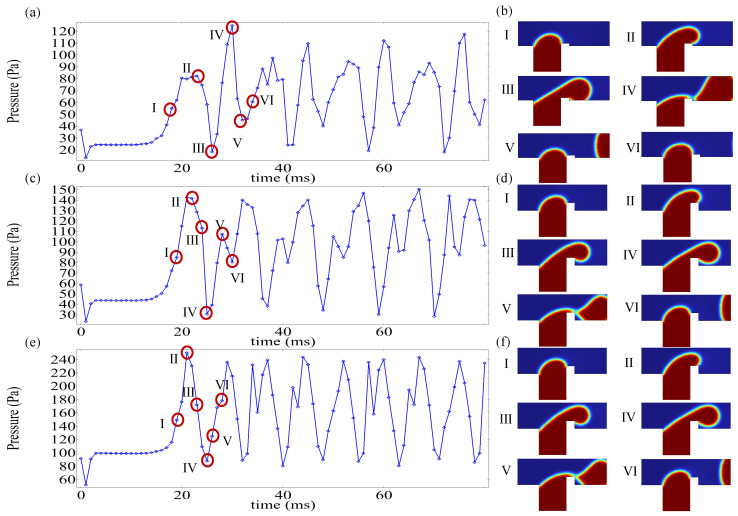
The evolution of the channel pressure drop over time under different rib height ratios ($a/w_{c}$) and the corresponding dispersed-phase morphology. (**a**,**b**) $a/w_{c}=0.1$; (**c**,**d**) $a/w_{c}=0.3$; (**e**,**f**) $a/w_{c}=0.5$.

Figure 13 presents the temporal evolution of mid-channel pressure drop with variations $a/w_{c}$. When the rib height ratio $a/w_{c}=0.1$, the pressure drop $P_{u}-P_{d}$ attains its first peak (82 Pa) at t = 23 ms, corresponding to State II in Figure 13b, where the dispersed phase traverses downstream post-rib passage. The droplet formation cycle measures 16 ms, with consistent dispersed-phase morphology between cycle boundaries (States I–VI). At the rib height ratio $a/w_{c}=0.5$, the peak pressure drop rises to 142 Pa at t = 22 ms, aligned with State II in Figure 13d, marking the dispersed phase’s passage over the rib apex. The formation cycle shortens to 11 ms. For the case of rib height ratio $a/w_{c}=0.5$, the maximum pressure drop surges to 250 Pa at t = 21 ms, corresponding to State II in Figure 13e during rib traversal. The cycle minimizes to 8 ms, representing a 50% reduction compared to that of $a/w_{c}=0.1$. Under constant flow rates and electric field strength, elevated rib heights accelerate droplet formation kinetics, enabling high-frequency generation of smaller droplets in ribbed T-junction microchannels.

### 4.2. Flow Diagram of the Dispersed Phase

The deformation and evolution of the two-phase interface are governed by a balance of three key forces: the shear force exerted by the continuous phase, the electric field force, and surface tension. The shear force and electric field force act synergistically to deform the dispersed-phase interface, whereas interfacial tension counteracts this deformation by minimizing the interfacial energy. The interplay between (${\mathrm{Ca}}_{e}$) and (Ca) defines distinct interfacial regimes that dictate force dominance, whether electric, viscous, or capillary forces prevail. These regimes critically influence the transition between flow diagrams observed in microchannels.

**Figure 14:**
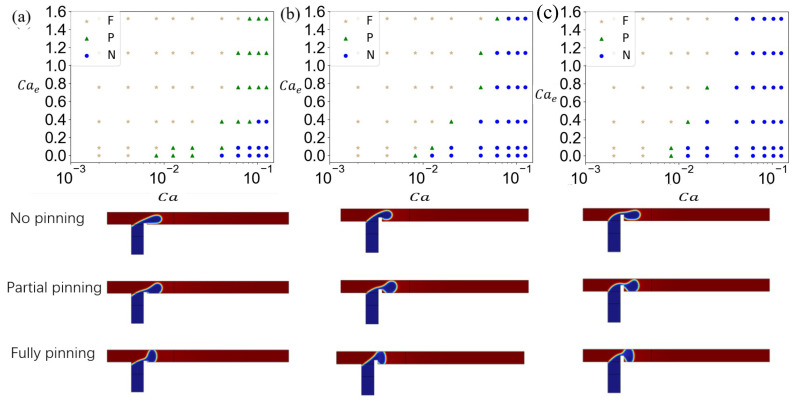
The flow state diagram of the dispersed phase in a T-junction microchannel with different rib height ratios and the corresponding flow behavior. (**a**) $a/w_{c}=0.1$, (**b**) $a/w_{c}=0.3$, and (**c**) $a/w_{c}=0.5$. Different symbols represent different flow states: stars (★) represent fully pinning (F), triangles (△) represent partially pinning (P), and circles (∘) represent unpinning (N).

As shown in Figure 14a, in the microchannel with a rib height ratio of $a/w_{c}=0.1$, when Ca<0.008, the fully pinning flow state is observed. The flow state of the dispersed phase transfers from partial pinning to fully pinning with the increase in ${\mathrm{Ca}}_{e}$. When 0.012<Ca<0.02, the dispersed phase exhibits partially pinning for ${\mathrm{Ca}}_{e}<0.09$; when ${\mathrm{Ca}}_{e}>0.38$, partially pinning transitions to fully pinning. In the flow field where Ca=0.041, progressive ${\mathrm{Ca}}_{e}$ increases induce sequential transitions from an unpinning pattern to partially pinning and finally to a fully pinning regime. When Ca>0.062, the unpinning regime vanishes and a fully pinning regime emerges at low ${\mathrm{Ca}}_{e}$. The change in the dispersed-phase flow state in Figure 14a is a result of the competition between the electric field force and the shear force of the continuous phase. At fixed ${\mathrm{Ca}}_{e}$, increasing Ca shifts the morphology from fully pinning to partially pinning and finally an unpinning state, underscoring the shear force of the continuous-phase dominance at higher Ca.

Figure 14b shows the dispersed-phase flow state diagram in a T-junction microchannel with a rib height ratio of $a/w_{c}=0.3$. Compared to $a/w_{c}=0.1$, the unpinning regime occupies a significantly expanded parametric domain. Notably, at Ca=0.012 and ${\mathrm{Ca}}_{e}=0$, the unpinning regime emerges, underscoring the dominance of hydrodynamic effects over electric field forces. This suggests that elevated rib heights and increased Ca synergistically amplify continuous-phase shear effects on the dispersed phase. A comparative analysis of Figure 14a,b reveals that increasing $a/w_{c}$ reduces the parametric domain of the partially pinning regime. The partially pinning regime, initially observed at high Ca and ${\mathrm{Ca}}_{e}$, fully transitions to the unpinning regime. This transition arises because the elevated rib lifts the dispersed phase, increasing its separation from the lower channel wall and thereby suppressing adhesion. At $a/w_{c}=0.5$ in Figure 14c, the dispersed-phase flow regime diagram demonstrates near-complete suppression of partially pinning states. While all three regimes persist, the partially pinned regime occupies a minimal parametric domain. At low Ca, reduced continuous-phase flow rates allow substantial dispersed-phase accumulation prior to necking, sustaining fully pinning regime dominance. As Ca increases, partially pinning domains convert into unpinning regimes. At high capillary numbers, the unpinning regime becomes ubiquitous. This is because the high $a/w_{c}$ elevates the dispersed phase, and also increasing continuous-phase shear force further inhibits adhesion.

### 4.3. Droplet Size of Microdroplets

**Figure 15:**
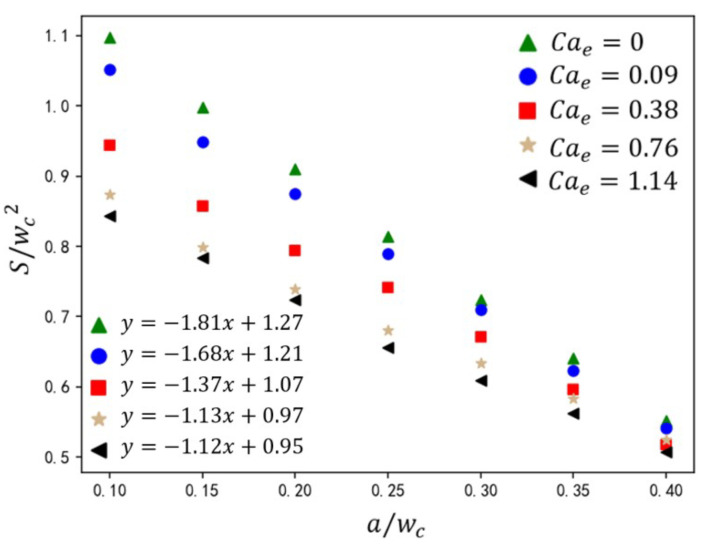
The effect of rib height ratio on droplet size under different electric capillary numbers. The straight-line equation in the lower-left corner represents the fitting formula for the calculated data under different electric capillary numbers, where *y* represents the droplet size $S/w_{c}^{2}$ and *x* represents the rib height ratio $a/w_{c}$.

Droplet size is one of the key aspects of droplet generation in T-junction microchannels. In this section, the effects of the electric capillary number (${\mathrm{Ca}}_{e}$), rib height ratio ($a/w_{c}$), dispersed-phase flow rate $Q_{d}$, and two-phase viscosity ratio λ on droplet size are studied. As shown in Figure 15, under inlet flow rate ratio Q=3 and capillary number Ca=0.036, the variation in droplet size $S/(w_{c}^{2})$ is shown for rib height ratios ($a/w_{c}=0.10–0.45$) and electric capillary numbers (${\mathrm{Ca}}_{e}=0–1.14$). The data demonstrate that increasing $a/w_{c}$ synergistically amplifies the shear stress exerted by the continuous phase on the dispersed phase, driving a monotonic reduction in normalized droplet size. This trend adheres to the linear scaling law:

(19) $$\frac{S}{w_{c}^{2}}=\omega \left(\frac{a}{w_{c}}\right)+\epsilon$$

where *S* is the area of dispersed phase, $w_{c}$ the width of the main channel inlet, *a* the rib height, and ω and ϵ the linear fitting parameters. Increasing ${\mathrm{Ca}}_{e}$ amplifies the slope ω of the linear fit while reducing the intercept ϵ, reflecting the reduction in stronger shear-driven droplet size. As shown in Figure 15, regardless of the height ratio of the ribs, the droplet size formed in the T-junction microchannel with the addition of an electric field is relatively smaller compared to the passive T-junction mode. For example, when the rib height ratio is 0.1 and there is no electric field, the effective size of the formed droplet is close to 1.1. When ${\mathrm{Ca}}_{e}$ = 1.14, the effective size of the formed droplet is 0.85. However, this ${\mathrm{Ca}}_{e}$-dependent sensitivity diminishes with increasing rib height ratio $a/w_{c}$. At a low $a/w_{c}$ regime, weak continuous-phase shear allows electric field forces to dominate. Rising ${\mathrm{Ca}}_{e}$ triggers morphology transitions from an unpinning to fully pinning state, accelerating necking dynamics and reducing droplet size. At a high rib height regime, rib-induced elevation displaces the dispersed phase from channel walls, suppressing adhesion. Continuous-phase flow acceleration dominates, sustaining unpinning flow regardless of ${\mathrm{Ca}}_{e}$. Consequently, ${\mathrm{Ca}}_{e}$ exerts minimal influence on droplet generation.

**Figure 16:**
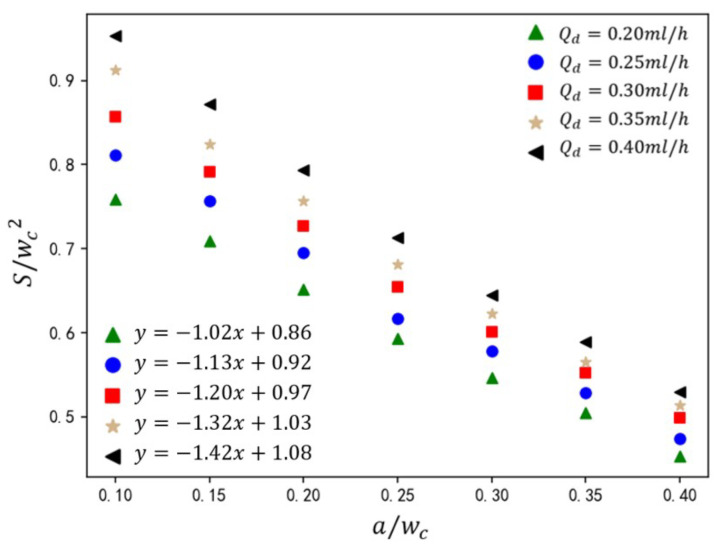
The effect of rib height ratio on droplet size under different dispersed-phase flow rates. The straight-line equation in the lower-left corner represents the fitting formula for the calculated data under different electric capillary numbers, where *y* represents the droplet size $S/w_{c}^{2}$ and *x* represents the rib height ratio $S/w_{c}^{2}$.

The velocity of the dispersed phase governs its volumetric occupancy within the main channel, directly influencing droplet size. As shown in Figure 16, under fixed dimensionless parameters (Ca=0.036, $Ca_{e}=1.14$), the normalized droplet size $S/(w_{c}^{2})$ is presented for rib height ratios $a/w_{c}=0.10–0.45$ and dispersed-phase flow rates $Q_{d}=0.2–0.4\;\mathrm{mL}/h$. Increasing dispersed-phase velocity elevates its volumetric influx into the main channel, augmenting neck thickness during droplet formation. This delays neck contraction, resulting in larger droplet sizes. At the constant value of $Q_{d}$, droplet size exhibits a linear inverse relationship with $a/w_{c}$. Higher ribs amplify shear thinning, accelerating neck rupture and reducing $S/(w_{c}^{2})$. From the analysis of Figure 16, it can be observed that empirical analysis of the five $Q_{d}$ cases yields the correlation

(20) $$\frac{S}{w_{c}^{2}}=\left(-0.62-1.8Q_{d}/Q_{c}\right)\left(\frac{a}{w_{c}}\right)+\left(0.64+0.99Q_{d}/Q_{c}\right)$$

This current formula can accurately predict the droplet size generated in a T-junction microchannel with varying rib heights under an electric field.

**Figure 17:**
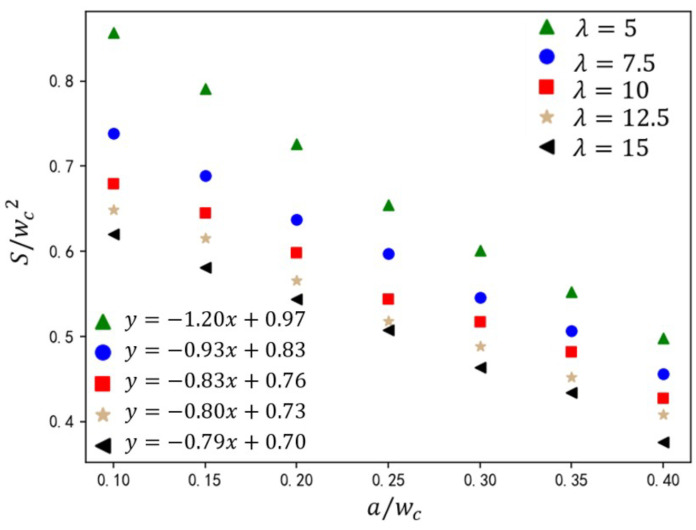
The effect of rib height ratio on droplet size under different viscosity ratios. The straight-line equation in the lower-left corner represents the fitting formula for the calculated data under different electric capillary numbers, where *y* represents the droplet size $S/w_{c}^{2}$ and *x* represents the rib height ratio $a/w_{c}$.

The viscosity ratio λ governs the interfacial velocity gradient, thereby modulating shear forces exerted by the continuous phase on the dispersed phase. As shown in Figure 17, under fixed operating conditions (Q=3 and $Ca_{e}=1.14$), the variation in droplet size is presented for rib height ratios $a/w_{c}=0.10–0.45$ and viscosity ratios λ=5–15. At the constant value of λ, $S/w_{c}^{2}$ exhibits a linear inverse correlation with $a/w_{c}$. Elevated ribs amplify shear via flow constriction, accelerating neck rupture. Increasing continuous-phase viscosity (higher λ) reduces droplet size $S/w_{c}^{2}$ due to enhanced shear thinning. At low λ regime (λ<8), droplet size sensitivity to viscosity changes is pronounced (size variation $\Delta S/w_{c}^{2}>12\%$). At high λ regime (λ>8), size variations diminish $\left(\Delta S/w_{c}^{2}<4\%\right)$, indicating shear dominance over viscous damping. This shear amplification suppresses viscosity-driven effects, narrowing droplet size differences across λ.

**Figure 18:**
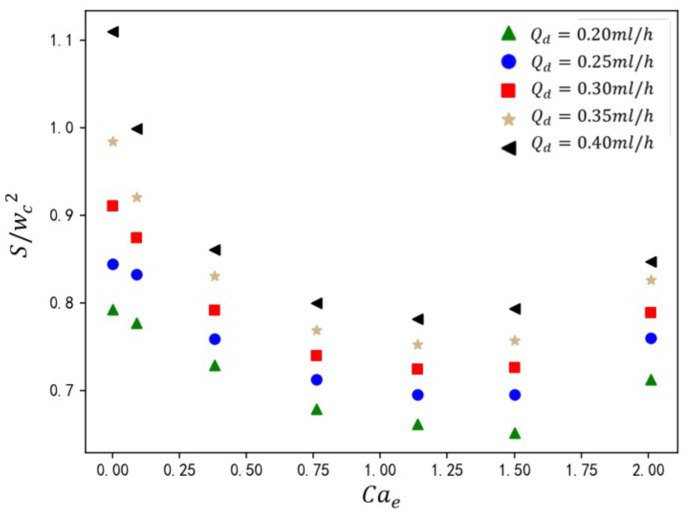
The effect of electric capillary number on droplet size at different dispersed-phase flow rates.

Under fixed geometric parameters (Ca=0.036, $a/w_{c}=0.20$, and $b/w_{c}=0.30$), the variation in droplet size is investigated under different dispersed-phase flow rates $Q_{d}=0.2–0.4\;\mathrm{mL}/h$ and electric capillary numbers $Ca_{e}=0–1.14$. As shown in Figure 18, droplet size $S/w_{c}^{2}$ first decreases, then increases with rising $Ca_{e}$. Morphology transitions from unpinning to partially pinning state, reducing neck stability and droplet size at low $Ca_{e}$ regime. Fully pinning morphology allows electric field forces to enhance dispersed-phase influx, increasing $S/w_{c}^{2}$ at high $Ca_{e}$ regime. At fixed $Ca_{e}$ values, $S/w_{c}^{2}$ scales linearly with $Q_{d}$. Thin dispersed-phase necks enable rapid pinch-off, yielding smaller droplets at low $Q_{d}$ regime. For high $Q_{d}$ regime, thickened necks prolong contraction time, increasing $S/w_{c}^{2}$.

**Figure 19:**
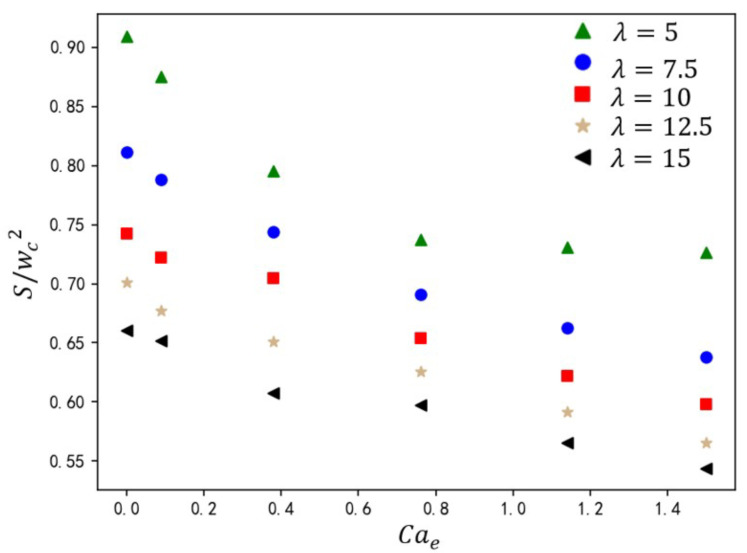
The effect of electric capillary number on droplet size at different viscosity ratios.

The interplay between viscosity ratio λ and electric field strength $Ca_{e}$ critically governs droplet formation dynamics in ribbed T-junction microchannels. Under fixed geometric and flow conditions (Q=3, $a/w_{c}=0.20$ and $b/w_{c}=0.30$), the variation in droplet size $S/w_{c}^{2}$ is studied for viscosity ratios λ=5–15 and electric capillary numbers $Ca_{e}=0–1.14$. As shown in Figure 19, for the fixed λ values, $S/w_{c}^{2}$ monotonically decreases with rising $Ca_{e}$. Increasing $Ca_{e}$ drives morphology transitions from unpinning to pinning state. Electric forces stabilize the dispersed phase, accelerating neck rupture and reducing droplet size. For the constant value of $Ca_{e}$, $S/w_{c}^{2}$ decreases with higher λ. Elevated λ enhances the shear stress of the continuous phase at the interface, shortening the droplet formation time.

## 5. Conclusions

This study investigates the mechanism of droplet generation in a ribbed T-junction microchannel under the influence of an electric field. By integrating active electric field control with passive rib structures, we propose a hybrid approach to precisely regulate the droplet size and droplet formation dynamics. The key findings of this study are summarized as follows:

1. The electric capillary number significantly influences the flow behavior of the dispersed phase, inducing three distinct flow regimes of the dispersed phase: unpinning, partially pinning, and fully pinning. As the electric capillary number increases, the contact area between the dispersed phase and the channel wall enlarges, enhancing droplet formation and reducing droplet size.

2. The rib structure plays a crucial role in modifying the shear stress distribution within the microchannel. As the rib height increases, the dispersed phase undergoes a progressive transition from a fully pinning state to an unpinning state. This results in a linear reduction in droplet size and an increase in droplet formation frequency.

3. A new empirical correlation is established to predict droplet size as a function of rib height and dispersed-phase flow rate.

(21) $$\frac{S}{w_{c}^{2}}=\left(-0.62-1.8Q_{d}/Q_{c}\right)\left(\frac{a}{w_{c}}\right)+\left(0.64+0.99Q_{d}/Q_{c}\right).$$

4. The effects of flow rate ratio and viscosity ratio on droplet formation were further examined. An increase in the flow rate ratio leads to increased influx of the disperse phase, precipitating a notable increase in the thickness of the neck region and the growth of the droplet size, whereas a higher viscosity ratio enhances the interfacial forces, leading to a reduction in droplet size.

These findings provide new insights into the interplay between passive structural modifications and the active control of the electric field of the droplet in microfluidic mechanics. Future research should systematically investigate the optimization of the rib structure and electrode placement in microfluidic droplet generation systems.

## Figures and Tables

**Table 1:** Geometric and physical parameters.

Parameter	Value
Width of main channel, $w_{c}$ (μm)	100
Width of side channel, $w_{d}$ (μm)	100
Length of main channel, *L* (μm)	1500
Length of side channel, L2 (μm)	250
Depth of channel (μm)	100
Density of CP, $\rho_{c}$ (kg/m^3^)	1000
Density of DP, $\rho_{d}$ (kg/m^3^)	1000
Dynamic viscosity of CP, $\mu_{c}$ (Pa·s)	0.005
Dynamic viscosity of DP, $\mu_{d}$ (Pa·s)	0.001
Surface tension, γ (N/m)	0.005
Dielectric permittivity of CP, $\varepsilon_{c}$	4.3
Dielectric permittivity of DP, $\varepsilon_{d}$	21.5
Conductivity ratio, $R=\sigma_{c}/\sigma_{d}$	0.1
Electric potential, *U* (V)	0–250

**Table 2:** Geometric and physical properties of the microchannel for data validations.

Parameter	Value
Width of main channel, $w_{c}$ (μm)	100
Width of side channel, $w_{d}$ (μm)	50
Depth of channel	33
Density of CP, $\rho_{c}$ (kg/m^3^)	1000
Density of DP, $\rho_{d}$ (kg/m^3^)	1000
Dynamic viscosity of CP, $\mu_{c}$ (Pa·s)	0.01
Dynamic viscosity of DP, $\mu_{d}$ (Pa·s)	0.001
Surface tension, γ (N/m)	0.0365

## Data Availability

The datasets generated and analyzed during the current study are available from the corresponding author upon reasonable request.
